# Mevalonate Kinase Deficiency and Neuroinflammation: Balance between Apoptosis and Pyroptosis

**DOI:** 10.3390/ijms141223274

**Published:** 2013-11-26

**Authors:** Paola Maura Tricarico, Annalisa Marcuzzi, Elisa Piscianz, Lorenzo Monasta, Sergio Crovella, Giulio Kleiner

**Affiliations:** 1 Department of Medicine, Surgery and Health Sciences, University of Trieste, Piazzale Europa 1, Trieste 34128, Italy; E-Mails: paola.tricarico@burlo.trieste.it (P.M.T.); sergio.crovella@burlo.trieste.it (S.C.); 2 Health Genetic Unit, Institute for Maternal and Child Health “Burlo Garofolo”, via dell’Istria 65/1, Trieste 34137, Italy; E-Mails: elisa.piscianz@gmail.com (E.P.); giulio.kleiner@burlo.trieste.it (G.K.); 3 Epidemiology and Biostatistic Unit, Institute for Maternal and Child Health “Burlo Garofolo”, via dell’Istria 65/1, Trieste 34137, Italy; E-Mail: lorenzo.monasta@burlo.trieste.it

**Keywords:** mevalonate, inflammation, neurodegeneration, apoptosis

## Abstract

Mevalonic aciduria, a rare autosomal recessive disease, represents the most severe form of the periodic fever, known as Mevalonate Kinase Deficiency. This disease is caused by the mutation of the *MVK* gene, which codes for the enzyme mevalonate kinase, along the cholesterol pathway. Mevalonic aciduria patients show recurrent fever episodes with associated inflammatory symptoms, severe neurologic impairments, or death, in early childhood. The typical neurodegeneration occurring in mevalonic aciduria is linked both to the intrinsic apoptosis pathway (caspase-3 and -9), which is triggered by mitochondrial damage, and to pyroptosis (caspase-1). These cell death mechanisms seem to be also related to the assembly of the inflammasome, which may, in turn, activate pro-inflammatory cytokines and chemokines. Thus, this particular molecular platform may play a crucial role in neuroinflammation mechanisms. Nowadays, a specific therapy is still lacking and the pathogenic mechanisms involving neuroinflammation and neuronal dysfunction have not yet been completely understood, making mevalonic aciduria an orphan drug disease. This review aims to analyze the relationship among neuroinflammation, mitochondrial damage, programmed cell death, and neurodegeneration. Targeting inflammation and degeneration in the central nervous system might help identify promising treatment approaches for mevalonic aciduria or other diseases in which these mechanisms are involved.

## 1. Introduction

**Figure 1.**
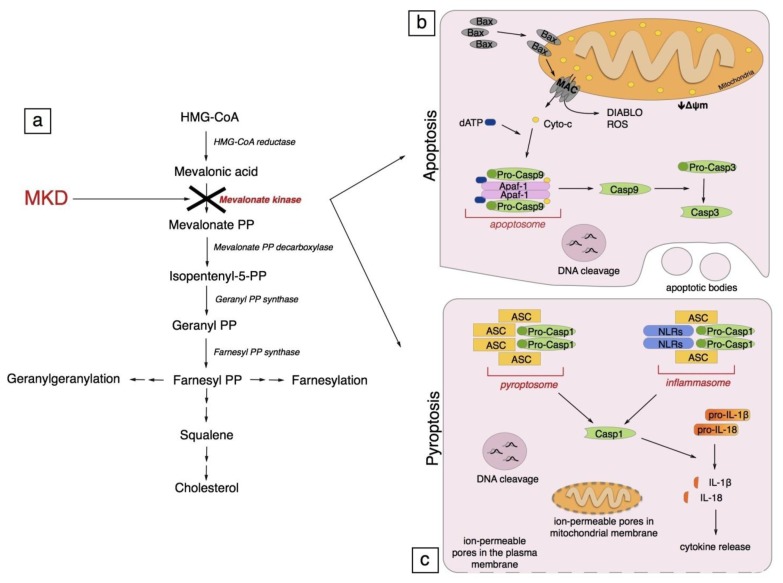
Mevalonate pathway and programmed cell death. (**a**) Mevalonate Kinase Deficiency (MKD) is characterized by a decrease of Mevalonate Kinase (MK, in red) residual activity. MK is the second enzyme of the mevalonate pathway. The programmed cell death occurring in MKD is linked to both apoptosis and pyroptosis pathways; (**b**) Intrinsic apoptosis pathway: BAX (Bcl-2-associated X protein) is activated and then, after oligomerization, it forms a channel into the mitochondria external membrane known as MAC (mitochondrial apoptosis-induced channel). MAC is important for the release of: cytochrome c, DIABLO (a second mitochondria-derived activator of caspase), ROS (reactive oxygen species), and for the dissipation of the mitochondrial transmembrane potential (Δψm). Cytochrome c binds to Apaf-1 (apoptotic protease-activating factor 1), forming the apoptosome that activates caspase-9 in an ATP-dependent manner. Active caspase-9 cleaves and activates effector caspase-3. Active caspase-3 cleaves target proteins that induce the cell death characterized by a DNA cleavage and the development of the membrane-enclosed apoptotic bodies; (**c**) Pyroptosis is a caspase-1 dependent programmed cell death. Caspase-1 can be activated by pyroptosoma and by inflammasome. Pyroptosoma is composed of oligomerized ASC (apoptosis-associated speck-like protein containing a CARD) dimers; inflammasome is composed of NLRs (nucleotide-binding oligomerization-domain protein-like receptors) and ASC, and both of them activate caspase-1. Active caspase-1 induces: maturation of pro-IL-1β and pro-IL-18 into, respectively, IL-1β and IL-18; DNA cleavage, and the formation of ion-permeable pores in the plasma membrane and in mitochondrial membrane.

Mevalonate Kinase Deficiency (MKD) is a rare, autosomic recessive, metabolic disease, caused by mutations in the *MVK* gene (12q24.11, NM_000431) coding for the enzyme mevalonate kinase (MK) (E.C. 2.7.1.36), the second enzyme of the mevalonate pathway, the route to cholesterol (Figure 1a) [1,2].

The severity of the disease is linked with the residual activity of MK; a residual activity between 1%–8% leads to a mild autoinflammatory phenotype, called hyper immunoglobulinemia D syndrome (HIDS, OMIM #260920), the symptoms of which are recurrent episodes of fever and associated inflammatory events. A residual activity below the level of detection leads to the most severe form of this pathology, known as mevalonic aciduria (MA, OMIM #610377), which, in addition, shows developmental delay, dysmorphic features, ataxia, cerebellar atrophy, psychomotor retardation, and sometimes death occurs in early childhood [3].

Although in the past decade the knowledge of the pathogenesis of MKD has increased, the link between genetic defect and phenotype is not yet clear. The most accredited pathogenic hypothesis is that the inflammatory phenotype is caused by shortage of isoprenoid compounds [4–6]. Thus far, this condition has been reproduced in biochemical experimental models obtained using drugs able to block the mevalonate pathway [7]. The so-caused cellular damage is driven by caspase-1 alone or in conjunction with caspase-3, via pyroptosis-apoptosis [8]. Recent data showed a pivotal role of the inflammasome platform at a systemic level [9], but its role in neurological impairments has never been ascertained. Finally, it has been found that the inflammasome involvement is deeply entangled with mitochondrial redox modulation [10–12].

MKD is considered today an orphan and neglected disease, and an aetiologic treatment is still unavailable. The attainment of new pharmacological treatments is of fundamental importance especially for the most severe forms of MKD, in which even the nervous system is involved.

## 2. Generalities on Mevalonate Pathway in the Central Nervous System

### 2.1. Neuroinflammation and the Mevalonate Pathway

Neuroinflammation plays with its action a fundamental role in the central nervous system (CNS), but, at the same time, it could exert both beneficial and detrimental effects on the nervous tissue. Indeed, a low-level, quick-ending and restrained inflammatory process is thought be neuroprotective, whereas the presence of a chronic process of inflammation could lead to negative effects.

The CNS has been considered for a long time an “immune privileged” area [13,14], even if this concept is still unclear and being studied. This privileged status is anatomically achieved due to the presence of the blood-brain barrier (BBB). The BBB is formed essentially by endothelial cells of brain capillaries in tight contact with astrocytes and pericytes, of which duties comprise to maintain the chemical balance and homeostasis within the CNS, to limit the passage of antibodies and immune cells into the brain, and to connect the neuronal environment with the periphery, in terms of transportation of selected substances in and out of the brain [15,16]. Thus, any dysfunction or disorder of this fundamental structure may lead to several diseases [17].

From an immunological point of view, CNS is also considered “immune privileged” due to the relative absence of specific antigen presenting cells [18] and the production of neuronal-supporting and anti-inflammatory molecules, such as TGF-α and IL-10 [19,20].

However, as said before, the “immune privileged” status of CNS is nowadays an important matter of study, since neurodegeneration is one of the most intriguing and challenging pathological condition in modern society. Indeed, despite these features, neuroinflammation occurs in many pathologies linked to CNS, such as neurodegenerative diseases, trauma and infections, and involves during its course several cellular and molecular components of innate and adaptive immunity [21].

The BBB has been discovered to have the ability to change its own permeability to cellular traffic according to molecular signaling of local-secreted cytokines and chemokines [22]. In addition, CNS hosts resident cells of innate immunity, microglia and astrocytes. Microglial cells represent the neuronal counter-part of macrophages, since they belong to the monocyte-macrophage system. They physiologically patrol and monitor the CNS in a quiescent state, exerting neuroprotective and neurogenetic actions, as well as homeostasis maintenance [23–25]. However, they have the capacity to be activated, proliferate, and assume an amoeboid shape when pathological changes occur [13,26]. Once activated, microglial cells gain macrophagic activity and upregulate MHC-II, absorbing debris and apoptotic cells and exposing antigens as antigen-presenting cells (APCs) [27]. Furthermore, they start to secrete proinflammatory cytokines, such as TNF-α, IL-6 and IL-1β, and chemokines, such as RANTES and MCP-1, in order to recruit professional APCs to the site of damage [28]. In physiological conditions, astrocytes are involved in the constitution of the BBB and serve as support to neurons, but they can also be activated in case of damage or inflammation and change their shape in order to generate a glial scar. As well as microglia, these cells are also able to release cytokines and chemokines, such as TNF-α, IL-6 and IL-1, and IFN-γ [29].

### 2.2. Cholesterol Metabolism in the Central Nervous System

Cholesterol metabolism at systemic levels is regulated by a fine balance between endogenous production, exogenous uptake, and, finally, by the exchange and transportation between several body districts mediated by apolipoproteins transported by the blood flow [30]. However, due to the presence of the BBB, the CNS is isolated from the turnover of systemic cholesterol and develops an autonomous mechanism for the maintenance of biosynthesis and homeostasis of this molecule. In the CNS, neurons and glia tightly cooperate in order to achieve this aim. Even if every cell type is able to synthesize it, it has been demonstrated that astrocytes produce from two- to three-fold more cholesterol than neurons [31] and deliver it to neuronal cells via apolipoproteins (ApoE, ApoD, ApoJ, ApoA1) [32]. The brain is the most cholesterol-rich region of the mammalian body, and this molecule is crucial to neurons, as it influences the synaptic function due its structural role in the synaptic membrane [33]. For this reason a perturbation of this metabolic pathway often leads to a pathological condition. As previously mentioned, almost all brain cholesterol is produced *in situ*, and, in case of a blockade of the pathway, compensatory mechanisms (for example, dietary intake) are lacking. Indeed, it has been seen that the imbalance in the CNS cholesterol homeostasis is often concurrent to several neurodegenerative diseases, even if the underlying mechanisms have not been discovered yet. Thus, the reduction of cholesterol levels seems to affect the synaptic transmission. Indeed, an intrinsic blockade of the mevalonate pathway caused by a genetic mutation in the developing brain of a newborn, may affect the entire growth of a physiological neuronal network, as it actually seems to occur in the most severe cases of MKD.

## 3. Models of MKD: *In Vitro* and *in Vivo*

The search of new drugs/therapies for MKD could take advantage of *in vitro* and *in vivo* models mimicking the characteristics of the human disease.

For ethical reasons, rather than on a MKD animal model, researchers focused first on a cellular model.

Indeed, a MKD model has been developed to reproduce the genetic deregulation on the mevalonate pathway using compounds, such as aminobisphosphonates and/or statins, known to inhibit different enzymes of this biochemical pathway (Figure 1a). This system led to a moderate inflammatory phenotype that could be amplified by the subsequent administration of a bacterial compound, such as muramyl dipeptide (MDP) or lipopolysaccharide (LPS) [34,35]. The role of these pro-inflammatory agents was to mimic the acute phase of the inflammatory process.

The cellular lines chosen for this purpose were primarily monocytes and macrophagic cells, as researchers wanted to study the inhibitory effect in the component of the immune system, which was involved in the inflammation response shown by MKD patients.

Using the same biochemical approach and mechanism, a biochemical mouse model was developed [36], showing that the inhibition of the mevalonate pathway, through the use of aminobiphosphonates, leads to a moderate inflammatory phenotype that could be amplified by the subsequent injection of MDP or LPS [5,37].

One of the main limit of this approach is represented by the absence of a system able to measure intermediate compounds levels before and after treatments; moreover, data obtained using biochemical models are hardly comparable to those obtained using biological samples derived from MKD patients.

Even if the findings presented in these MKD models should be considered as preliminary, due to the adoption of a biochemical model of MKD, they should be taken as a first step in research aimed at better disclosing the MKD pathogenesis.

At present, a mouse model of MKD has been created with the deletion of one *MVK* [38], resulting in MKD phenocopy without the features of neurological dysfunction. Other reports on deletion of specific genes in cholesterol synthesis in the mouse model have revealed a high degree of embryonic lethality [39,40]. The lack of neurological involvement was the main weakness of this model, because it represents a crucial aspect of the most severe form of MKD disease.

It is important to say that cell lines from MKD patients do not exist: the anatomic evaluations of the neurological impairment of MKD patients have always been done and can be done only post-mortem. However, recently, a neuronal model of MKD has been developed in order to deepen the pathogenesis of this aspect of mevalonic aciduria [41].

## 4. Pathogenesis of Mevalonate Kinase Deficiency

The block of the cholesterol pathway in MKD, due to mutations in mevalonate kinase gene, causes a shortage of downstream metabolites [42,43].

While the excess of mevalonate is excreted via the kidneys in the urine, a shortage of downstream compounds is observed. Farnesyl pyrophosphate (FPP) is the branch point metabolite of the mevalonate pathway from which several biosynthetic routes depart. Of these pathways, one leads to squalene, cholesterol, and correlated metabolites, one leads to dolichol, polyprenyl chains of Heme A and ubiquinone, and one leads to geranylgeranyl pyrophosphate (GGPP) (Figure 1a).

In MKD patients not all these pathways are affected equally. Indeed, serum cholesterol remains within normal levels, as well as hepatic squalene and dolichol. On the contrary, a number of lowered metabolites of the mevalonate pathway are involved in protein prenylation.

Most proteins from the small GTPase superfamily are prenylated, and alterations in this post-translational mechanism could lead to altered localization or activation when isoprenoids are lacking [4,44].

It has been demonstrated that the lack of isoprenoids is associated with a decreased inflammation threshold, and conversely that exogenous isoprenoids can neutralize the pro-inflammatory phenotype both in *ex vivo* monocytes of MKD patients and in murine models [5,6]. A possible link between the lack of isoprenoids and inflammation could be explained by the overactivity of GTPases, which causes an activation of inflammasome and subsequently of caspase-1. Caspase-1 is required for the activation of IL-1β, maybe the most important pro-inflammatory cytokine in MKD.

Inflammasomes are cytoplasmic multi-protein complexes that function as sensors of endogenous or exogenous PAMPs (pathogen-associated molecular patterns). They are composed of one of several nucleotide-binding oligomerization-domain protein-like receptors (NLRs), including NLRP1, NLRP3, NLRP6, and NLRPC4. On sensing the relevant signal, they assemble, typically together with an adaptor protein, an apoptosis-associated speck-like protein (ASC) or a caspase activating and recruitment domain 8 (CARD8), into a multi-protein complex that governs caspase-1 activation and subsequent cleavage of effector pro-inflammatory cytokines, including pro-IL-1β and pro-IL-18. In addition, the function of mitochondria can be impaired, due to altered prenylation of Heme A and ubiquinone. Alteration in mitochondria function leads to release of ROS and other molecules capable to activate once more the inflammasome. Moreover, it has been recently demonstrated that mitochondrial ROS can activate NLRP3 and re-localize it to the mitochondrial membrane, through microtubule transport, where it is able to form a functional inflammasome with ASC and caspase-1 [10,45,46].

Activation of inflammasome with release of IL-1β causes attacks of fever in MKD patients, but, at a cellular level, it leads to an increase in cell death that resemble the mechanism of pyroptosis. IL-1β release is a complicated event, which is influenced by cell type and by the inflammatory stimulus. It has been demonstrated that activation of caspase-1 may need different stimuli to induce the activation of inflammasome in macrophages or glial cells, with regards to ATP-dependent mechanisms [47,48].

This could explain why pharmacological therapies can exert different degrees of control over the inflammation phenotype, while the neurological involvement, particularly severe in MA, remains the most important goal to be reached. Moreover, patients carrying same mutations on the *MVK* gene often exhibit a high variability of symptoms and, consequently, also of responses to the therapies. Although it is not established yet, this high variability could be explained by other genes or by the genetic background of patients, which contribute to the development of MKD.

## 5. Programmed Cell Death

MKD models, *in vitro* and *in vivo*, showed that the typical programmed cell death occurring in Mevalonate Kinase Deficiency is linked both to the intrinsic apoptosis pathway and to pyroptosis [41,49].

### 5.1. Apoptosis

#### 5.1.1. Generalities on Apoptosis

Apoptosis is an active programmed cell death and is described as immunologically silent. It is characterized by activation of cysteinyl aspartate-specific proteases or caspases that are divided into caspase initiators and effectors, depending on their point of entry into the apoptotic pathway. The intrinsic apoptosis pathway, also known as mitochondrial pathway, is activated by the initiator caspase-9 and by the effector caspase-3 [50].

The intrinsic pathway is activated by various intracellular stress conditions including DNA damage, cytotoxic insult, oxidative stress, infection, and cellular homeostasis. These various stimuli act by inhibiting or activating particular anti- or pro-apoptotic Bcl-2 family members [51,52].

The Bcl-2-associated X protein (Bax) is a very important pro-apoptotic protein. During cell stress, the inhibition of Bax is relieved, thus, promoting its oligomerization and the formation of channels into the mitochondria, through which cytochrome c is released into the cytosol [53]. Subsequently, cytochrome c binds to apoptotic protease-activating factor 1 (Apaf-1), forming the apoptosome that activates caspase-9 in an ATP-dependent manner. Active capase-9 cleaves and activates effector caspase-3. Caspase-3 then cleaves target proteins to trigger programmed apoptosis cell death characterized by cytoplasmic and nuclear condensation, DNA cleavage and by the development of the membrane-enclosed apoptotic bodies [52,54].

The Bax-induced channel formed into the mitochondria, known as the mitochondrial apoptosis-induced channel (MAC), besides allowing the release of cytochrome c, is important for: (a) the dissipation of the mitochondrial transmembrane potential (Δψm); (b) the release of other proteins into the cytosol, such as a second mitochondria-derived activator of caspase (SMAC), also known as DIABLO; and (c) the inhibition of the respiratory chain that cause the ROS overproduction (Figure 1b) [55–58]. All these processes of mitochondrial damage are also supported by the involvement of other Bcl-2 family members that modulate the existing mitochondrial permeability transition pore complex (PTPC) [59].

These mitochondrial dysfunctions represent a central causal factor in the pathogenesis of neurodegenerative diseases such as Alzheimer’s disease, Parkinson’s disease, Huntington’s disease, and many others [8,59,60]. In recent years, researchers focused on the identification of compounds able to block the mitochondrial damage and, subsequently, neuronal death.

#### 5.1.2. Apoptosis in Mevalonate Kinase Deficiency

Apoptosis, in particular via the mitochondrial pathway, plays a very important role in MKD.

At systemic level the cells, in particular monocytes, follow caspase-3 dependent apoptosis. This process was observed both in primary human monocytes from MKD and in biochemical MKD models obtained from monocyte cell lines [61,62].

In patients with severe forms of MKD, magnetic resonance imaging revealed progressive cerebellum atrophy that seems to be a direct consequence of neuronal apoptosis [63]. This is confirmed in biochemical MKD models obtained in neuroblastoma cell lines treated with statin, one inhibitor of the mevalonate pathway. The block of the pathway causes an increased apoptosis sustained by the activation of caspase-3 and caspase-9, and a mitochondrial dysfunction that plays a crucial and important role in the pathogenesis of MKD [41,48]. On the contrary, Evelien J. Bodar *et al.*, in 2007, observed that hereditary periodic fever syndromes are characterized by a defect in apoptosis regulation. In fact, they suggest that a defect in apoptosis regulation is the main trigger of the inflammatory response in these syndromes [64].

### 5.2. Pyroptosis

#### 5.2.1. Generalities on Pyroptosis

Pyroptosis, also known as caspase-1 dependent programmed cell death, is another form of programmed cell death. Unlike apoptosis, pyroptosis is not immunologically silent, as it involves the release of inflammatory cytokines IL-1β and IL-18 [65,66].

Pyroptosis has been initially described in monocytes, macrophages and dendritic cells infected with a range of microbial pathogens. It has also been observed in non-macrophage cells after non-infective stimuli, such as damage-associated molecular pattern molecules [67,68].

The pyroptotic activation of caspase-1 may be due to a multiprotein platform known as the inflammasome [69]. Another mechanism of caspase-1 activation requires the involvement of ASC pyroptosome, composed of oligomerized ASC (apoptosis-associated speck-like protein containing a CARD) dimers [69,70].

In both cases, active caspase-1 induces the formation of discretely sized ion-permeable pores in the plasma membrane [71]. These pores act on mitochondrial membrane potential and plasma membrane integrity, causing a dissipation of cellular ionic gradient, producing an increase osmotic pressure, water influx, cell swelling, release of cytokines, and, ultimately, cell lysis [72,73].

Furthermore, active caspase-1 catalyzes the proteolytic maturation of pro-IL-1β and pro-IL-18 into, respectively, IL-1β and IL-18. These cytokines are powerful mediators of inflammation that stimulate the production of secondary cytokines (Figure 1c) [65].

The pyroptotic pathway is characterized by a mitochondrial dysfunction. In this case, the mitochondrial damage is not the central causal factor of cellular death, it is only its consequence.

The caspase-1 activation, and consecutive cell death, plays a predominant role in the immune and central nervous systems and, in the brain, caspase-1 may directly regulate neuronal cell death in response to diverse insults [74–76].

#### 5.2.2. Pyroptosis in Mevalonate Kinase Deficiency

In literature, only few studies focus on the role of pyroptosis in MKD. However, the researchers substantially agree in suggesting pyroptosis as a fundamental step in inducing the inflammatory phenotype of MKD patients. Studies performed both on cell lines, in which the mevalonate pathway was biochemically blocked, and on MKD-patients-derived cells revealed a hyperproduction of IL-1β and IL-18, supposedly due to an overactive caspase-1 [8,35,77]. A hypothesis is that pyroptosis, triggered by the hyperactivity of caspase-1, may be caused by the lack of isoprenoid compounds, intermediates of the mevalonate pathway.

IL-1 family and the mechanisms that lead to its activation, such as caspase-1 and inflammasomes, are crucial events even in neuronal damage. Several studies show how inflammatory processes occur after acute brain insults, as well as in chronic neurodegenerative diseases [78,79]. For this reason, some researchers suggest that the neuronal death seen in the most severe form of MKD may indeed be caused by pyroptosis, in addition to apoptosis [40].

## 6. Oxidative Stress and Mitochondrial Damage through the Mevalonate Pathway

Oxidative stress is characterized by the increase in reactive species and it is involved in the development of many degenerative diseases. Reactive oxygen species (ROS) are the most representative reactive species in the cells, and they mainly are originated by mitochondrial respiration. ROS have been implicated in many inflammatory diseases; in particular, Familial Mediterranean fevers are characterized by an increase of mitochondrial ROS [79–81].

When ROS are highly concentrated, they react with protein, lipid, nucleic acid and carbohydrates inducing irreversible alterations. ROS appear to play a role in activating NLRP3 inflammasome, which in turn activates caspase-1 and pro-inflammatory cytokines [10]. ROS overproduction is a consequence of mitochondrial dysfunction related to apoptosis, and in particular to neuronal apoptosis.

In neurons, mitochondria participate in a variety of processes and play an essential role for providing energy and calcium buffering, required for synaptic transmission. In fact, mitochondrial damage is a central causal factor in the pathogenesis of neuroinflammation and neurodegeneration.

Mitochondrial dysfunction could be the link between neuroinflammation and neuronal degeneration. Dissipation of the mitochondrial transmembrane potential, the base of mitochondrial damage, is the most accredited cause of NALP3-inflammasome activation and subsequently release of pro-inflammatory cytokines. Similarly, this mitochondrial damage is the primary fundamental event of neuronal-programmed cell death.

Mevalonate pathway is important for the production of antioxidant molecules such as ubiquinone, heme A, selenoproteins and glutathione peroxidase.

Ubiquinone and heme A are involved in electron transport. Selenoproteins are important in development, metabolic homeostasis, and antioxidant defense. Glutathione peroxidase plays an important role in redox balance [82,83].

The block of the cholesterol pathway in MKD causes a shortage of these metabolites. In fact, a recent hypothesis is that the pathogenesis of MKD might be mainly explained by the oxidative stress related to the decrease of antioxidant molecules [83].

## 7. Concluding Remarks

Programmed cell death plays a crucial role in the inflammatory phenotype of MKD pathogenesis, and it has been demonstrated both *in vitro* and in animal models. Both apoptosis and pyroptosis are involved in the pathogenetic mechanism that sustains the secretion of IL-1β, the major marker of MKD, although, at present, the link between PCD and cytokines secretion remains unclear and PCD was evaluated both at system and cerebral levels in MKD models.

Moreover, recently published data have shown that the oxidative stress and mitochondrial damage act as a final trigger for PCD.

These new insights open a new frontier in the research of a pharmacological treatment for this orphan disease, by identifying new specific molecular targets, particularly for the treatment of its neurological aspects.

In conclusion, great improvements have been obtained in recent years in the field of MKD thanks to the identification of the molecular mechanisms involved in the pathogenesis of the disease, and in particular of the role of the PCD.

Although further studies are necessary, in our opinion a possible future application of these new findings could be the development of therapeutic strategies that will target the mitochondrial mechanism involved in MKD pathogenesis.
